# p62/sequestosome-1 as a severity-reflecting plasma biomarker in Charcot–Marie–Tooth disease type 1A

**DOI:** 10.1038/s41598-024-61794-w

**Published:** 2024-05-14

**Authors:** Byeol-A Yoon, Young Hee Kim, Soo Hyun Nam, Hye-Jin Lee, Seong-il Oh, Namhee Kim, Kyeong-Hee Kim, Young Rae Jo, Jong Kuk Kim, Byung-Ok Choi, Hwan Tae Park

**Affiliations:** 1 Peripheral Neuropathy Research Center (PNRC), Department of Translational Biomedical Sciences, Graduate School of Dong-A University, Busan, 49201 Republic of Korea; 2 Department of Neurology, Dong-A University College of Medicine, Busan, 49201 Republic of Korea; 3 Department of Molecular Neuroscience and Translational Biomedical Sciences, Dong-A University College of Medicine, Busan, 49201 Republic of Korea; 4 Department of Health Sciences and Technology, SAIHST, Sungkyunkwan University, Seoul, 06351 Republic of Korea; 5 Department of Neurology, Kyung Hee University Hospital, Kyung Hee University College of Medicine, Seoul, 02447 Republic of Korea; 6 Department of Laboratory Medicine, Dong-A University College of Medicine, Busan, 49201 Republic of Korea; 7 Department of Neurology, Samsung Medical Center, 81 Irwon-Ro, Gangnam-Gu, Seoul, 06351 Republic of Korea

**Keywords:** Neuroscience, Neurology

## Abstract

Autophagy is a self-degradation system for recycling to maintain homeostasis. p62/sequestosome-1 (p62) is an autophagy receptor that accumulates in neuroglia in neurodegenerative diseases. The objective of this study was to determine the elevation of plasma p62 protein levels in patients with Charcot–Marie–Tooth disease 1A (CMT1A) for its clinical usefulness to assess disease severity. We collected blood samples from 69 CMT1A patients and 59 healthy controls. Plasma concentrations of p62 were analyzed by ELISA, and we compared them with Charcot-Marie-Tooth neuropathy score version 2 (CMTNSv2). A mouse CMT1A model (C22) was employed to determine the source and mechanism of plasma p62 elevation. Plasma p62 was detected in healthy controls with median value of 1978 pg/ml, and the levels were significantly higher in CMT1A (2465 pg/ml, *p* < 0.001). The elevated plasma p62 levels were correlated with CMTNSv2 (*r* = 0.621, *p* < 0.0001), motor nerve conduction velocity (*r* =  − 0.490, *p* < 0.0001) and disease duration (*r* = 0.364, *p* < 0.01). In C22 model, increased p62 expression was observed not only in pathologic Schwann cells but also in plasma. Our findings indicate that plasma p62 measurement could be a valuable tool for evaluating CMT1A severity and Schwann cell pathology.

## Introduction

Charcot–Marie–Tooth disease (CMT) is the most common inherited peripheral neuropathy affecting one out of 2500 individuals^1^. Most patients with CMT develop motor weakness, sensory loss, and areflexia. Clinical outcome assessments, including the CMT neuropathy score version 2 (CMTNSv2), have been used to measure disease severity and progression in patients^2^. Recent studies have revealed that blood biomarkers for evaluating the pathology of peripheral axons and Schwann cells (SCs), the myelin-forming peripheral glia, appear to be useful for predicting disease severity in CMT. For example, axon-derived neurofilament light chain levels are elevated in the blood of patients with CMT patients who presenting high CMTNS^3,4^. It has recently been shown that neural cell adhesion molecule 1, transmembrane protease serine 5, and growth/differentiation factor-15 derived from pathologic SCs are elevated in the sera of CMT patients^5–7^. To extend the clinical usage of potential serum/plasma biomarkers for monitoring CMT progression, further development of biomarker targets with larger cohort studies is necessary.

Autophagy is a regulated lysosome-dependent degradation process that removes unnecessary and abnormal cellular components in cells^8^. Macroautophagy (hereafter written as autophagy) is the main pathway of autophagy during which damaged cell organelles (the cargo) are segregated into double-membrane autophagosomes, and the contents within autophagosomes are digested by fusion with lysosomes (autophagy flux)^9,10^. The recognition of cargo by autophagosomes requires the interaction of autophagy receptor proteins, such as p62/sequestosome-1 (p62) and optineurin, with cargo, and then both cargo and p62 are degraded by lysosomal hydrolases during autophagy flux^11,12^. Thus, the unusual accumulation of p62 in cells is a reliable indicator of dysfunction in autophagy flux^13^. It has recently been shown that dysfunction of lysosomes or autophagic flux results in the secretion of autophagosomal contents via extracellular vesicles, such as exosome, with autophagy machineries^14^. In line with this, extracellular p62 was found in the blood of patients with pathological condition^15^. In CMT1A, the most common inherited demyelinating neuropathy caused by overproduction of peripheral myelin protein 22, pathological SCs show a defect in autophagy with accumulation of ubiquitinated cargoes and p62 within cytoplasm^16,17^. Therefore, it is tempting to speculate that secretory p62 could be a potential target of blood biomarkers in CMT1A patients^18^.

Considering the secretion of p62 in pathological conditions^15^in this study, we performed ELISA to measure plasma p62 levels in patients with CMT1A and age-matched healthy controls (HC). Moreover, we not only investigated correlations of plasma p62 concentrations with various clinical parameters of CMT1A patients but also tried to validate our results in an animal model of CMT1A^2^.

## Materials and methods

### Participants and study designs

We consecutively enrolled 69 CMT1A patients between 2020 and 2021 after obtaining approval from Inherited Neuropathy Clinic at Department of Neurology, Samsung Medical Center (Seoul, South Korea). Written informed consent was obtained from all participants according to the protocol approved by the Institutional Review Board of the Samsung Medical Center at Sungkyunkwan University (SMC, 2020-01-146). *PMP22* duplication causing CMT1A was determined by hexaplex microsatellite PCR for the chromosome 17p12 region. All CMT1A patients in this study had a confirmatory causative genetic mutation (Table 1). Disease severity was measured using CMTNSv2^2^. Patients with CMT1A were divided into three groups according to disease severity; mild (CMTNSv2 ≤ 10), moderate (CMTNSv2, 11–20), and severe (CMTNSv2 ≥ 21).

**Table 1:** Demographic data, median p62 plasma concentrations, CMTNSv2 scores of the CMT1A patients, and healthy control.

	CMT1A	Healthy control
Number	69	59
Age (year) (SEM)	47.1 (1.55)	45.2 (1.15)
Sex, F/M	34/35	30/29
Duration (SEM)	21.3 (2.09)	NA
p62, pg/mL (IQR)	2465 (1812–3836)	1978 (1139–2522)
CMTNSv2 (IQR)	13 (8–18)	NA

CMTNSv2 = Charcot–Marie–Tooth neuropathy score version 2; CMT1A = *PMP22* duplication; SEM = standard error of the mean; IQR = interquartile range; NA = not applicable.

For plasma collection from HC, we enrolled participants who visited for a health check-up and were matched in sex and age to the CMT1A group. Those who had any neurological symptoms or medical history-based neurologic diseases were excluded. And all methods were performed in accordance with the relevant guidelines and regulations.

### Blood sample collection and storage

Blood samples were collected and processed within one hour. Blood was collected into EDTA tubes and centrifuged at 3000 rpm for 10 min at 4 °C. Plasma was then aliquoted and stored at − 80 °C.

### p62 measurements

Plasma p62 was measured using commercially available ELISA kits (LSbio, LS-F49427, Seattle, USA). All samples were analysed three times without dilution according to the manufacturer's instructions. The average repeatability coefficient of variation of a sample with a mean p62 concentration 2738 pg/ml was 7.31% and the interassay coefficient of variation was 8.78%. The limit of detection, determined as the mean blank signal + 3 standard deviation for the p62 assay, was 51.19 pg/ml. The lower limit of quantification, determined as the mean blank signal + 10 SD, was 173.5 pg/ml. All samples with p62 detection below the lower limit of quantification were considered to have a concentration of 0.

### Animals

All animal experiments were performed according to the protocol approved by the Dong-A University Committee on Animal Research (No. DIACUC 21-8) which follows the guidelines for animal experiments that were established by the Korean Academy of Medical Sciences. And this study is reported in accordance with ARRIVE guidelines.

C22 mice [B6;CBACa-Tg(*PMP22*)C22Clh/H] were obtained from the Samsung Medical Center (Seoul, Korea). The mouse model contained seven copies of the human *PMP22* gene leading to a demyelinating neuropathy ^19–21^.

### Immunofluorescent (IF) staining

Control C57BL/6 mice and C22 mice were perfusion-fixed with 4% paraformaldehyde in phosphate-buffered saline (PBS) after anesthesia with a mixture of 10% ketamine hydrochloride (Sanofi-Ceva, Düsseldorf, Germany; 0.1 ml/100 g body weight) and Rompun (Bayer, Leverkusen, Germany; 0.05 ml/100 g body weight). The sciatic nerves were obtained and then cryoprotected in a 20% sucrose solution. For analysis of p62 expression, cross sections of 10 μm thickness were made using a cryocut and stored in a deep freezer until further use. The slides were blocked with PBS containing 0.2% Triton X-100 and 5% bovine serum albumin for 1 h at room temperature. Next, the slides were incubated with primary antibodies overnight at 4 °C, and then washed three times with PBS. Thereafter, the slides were incubated with Cy3- or Alexa 488-conjugated secondary antibody at room temperature for 3 h, and stained with DAPI for 30 min. For light microscopic analysis, the stained sections were examined under an ApoTome fluoromicroscope with a Zeiss AxioImager 2 or a confocal microscope (ImageXpress or LSM800, Carl Zeiss, Göttingen, Germany).

### Antibodies used for immunofluorescence staining and western blot analysis

Antibodies against p62, glyceraldehyde 3-phosphate dehydrogenase, and myelin basic protein were obtained from Abcam (Cambridge, UK). Antibodies against medium-size neurofilament chain (NF-M) and β-actin were obtained from Thermo Fisher Scientific (Waltham, MA, USA) and Santa Cruz Biotechnology (CA, USA), respectively. Horseradish peroxidase-linked anti-rabbit IgG was purchased from Cell Signaling Lab (Beverly, MA, USA). Alexa Fluor 488 or Cy3-conjugated secondary antibodies were purchased from Molecular probes (Carlsbad, CA, USA).

### Western blot analysis

For western blot analysis, sciatic nerves were grossly dissected into the small fragments, and the tissue lysates were made using TissueLyser LT (Qiagen) in modified RIPA buffer containing 1% Triton X-100 in Tris–EDTA solution. RIPA lysates were centrifuged at 9000 g for 10 min at 4 °C, and the supernatant was collected. The proteins (10–35 μg) were separated by SDS-PAGE, and then transferred onto a nitrocellulose membrane (Amersham Biosciences). After blocking with 5% nonfat drymilk in Tris-buffered saline with Tween-20 (TBST; pH. 7.2) at room temperature for 1 h, membranes were incubated with primary antibodies (1:500–2000) in TBST containing 1% nonfat dry milk at 4 °C overnight. After three washes with TBST, membranes were incubated with a horseradish peroxidase-conjugated secondary antibody for 1 h at room temperature. Chemiluminescent reactions were performed using an enhanced chemiluminescence western blotting detection system (GE Healthcare). Images were then detected using Luminogragh 3 and quantified using a CS Analyser 4 (ATTO). Quantification was performed using a density analyser (CS Analyser 4), and the values were obtained from three independent experiments.

### Statistical methods

The results were expressed as the median ± standard error of the mean. ELISA data were analysed using one-way analysis of variance followed by the Kruskal–Wallis test and Sidak's multiple comparisons test using GraphPad Prism version 9 (GraphPad Software Inc., La Jolla, CA). Correlations were assessed using Spearman’s and Pearson’s correlation coefficients, and comparisons of plasma p62 concentrations between the two groups were analysed using the Mann–Whitney U test. Logistic regression and receiver operating characteristic (ROC) curve analyses were used to assess the diagnostic performance of plasma p62 in CMT1A patients.

## Results

### High plasma p62 level was observed in CMT1A patients

A total 69 CMT1A patients and 59 HC were enrolled in this study. Table 1 summarises the baseline demographics and clinical features, including disease duration, levels of plasma p62, and clinical scores of CMT1A patients. There was no significant difference in age and sex between the CMT1A and HC groups (*p* > 0.05).

**Figure 1:**
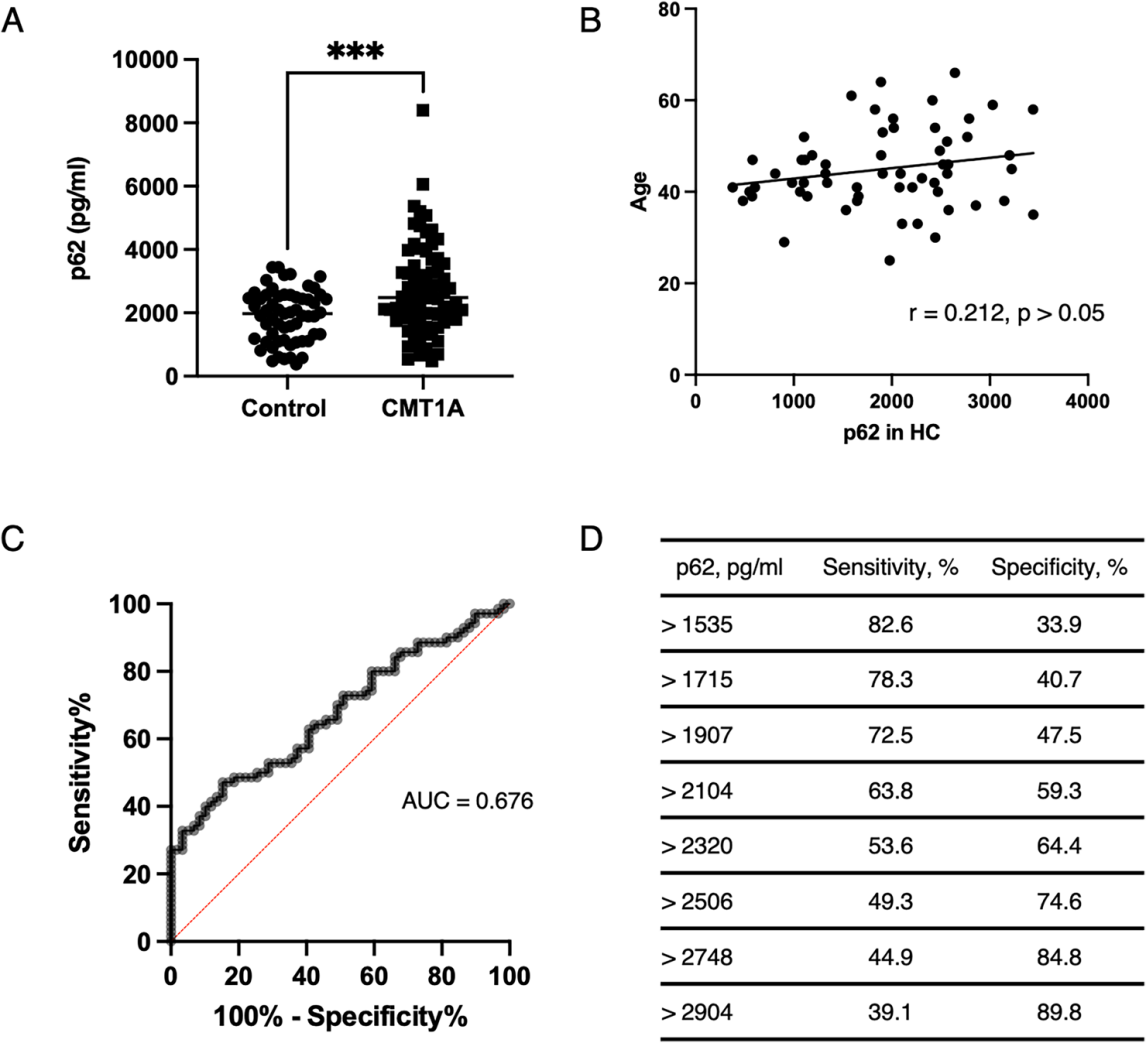
Plasma p62 concentrations were elevated in patients with Charcot–Marie–Tooth disease 1A patients. (**A**) Plasma p62 concentrations measured by ELISA in Charcot–Marie–Tooth disease (CMT) 1A patients and healthy controls (HC) (*** = *p* < 0.001). (**B)** Plasma p62 concentrations was not correlated with age in HC. (**C**) Receive operator characteristic (ROC) curve for prediction of CMT1A from HC based on the plasma p62 levels measured by ELISA. Area under the ROC curve was 0.676. (**D**) Sensitivity and specificity were assessed to determine the range of cut-off for detecting CMT1A patients.

ELISA results revealed that the median plasma p62 concentration was 1978 pg/ml in HC while CMT1A patients exhibited higher plasma levels of p62 (median, 2465 pg/ml, *p* < 0.01; Table 1, Fig. 1A) than that of HC. Statically significant differences were observed, but there was a great overlap between HC and CMT1A patients in p62 levels. Plasma p62 concentration was not correlated with age in HC (*r* = 0.212, *p* > 0.05) (Fig. 1B). Next, we plotted a receiver operator curve of plasma p62 concentration to determine how accurately we could predict CMT1A by evaluating plasma p62 levels. The calculated area under the curve was 0.676 (Fig. 1C). Plasma p62 concentration of 2104 pg/ml was found to be the best cut-off value to differentiate between patients with demyelinating CMT neuropathy and HC (Fig. 1D).

### Plasma p62 concentration reflected disease severity and duration of CMT patients

**Figure 2:**
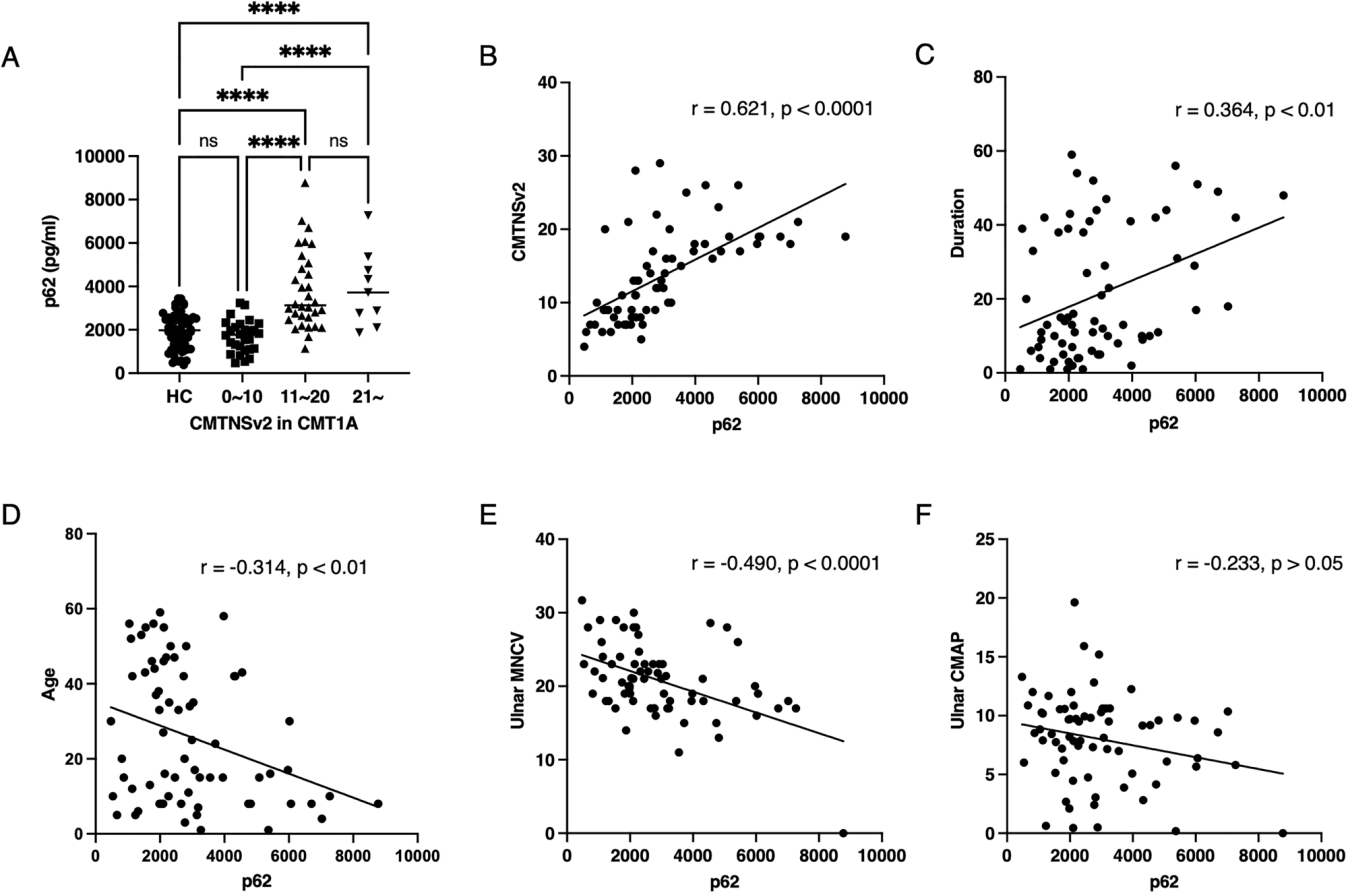
Plasma p62 concentration reflected disease severity and duration in Charcot–Marie–Tooth disease 1A patients. (**A**) Plasma p62 levels assessed according to mild, moderate, and severe groups in CMT1A patients (**** = *p* < 0.0001; ns = not significant). (**B**) and (**C**) Pearson coefficient analysis related to plasma p62 concentration, CMTNSv2, and disease duration in CMT1A patients. (**D**) Onset age was negatively correlated with plasma p62 level. (**E**) Ulnar motor nerve conduction velocities were negatively correlated with plasma p62 levels in CMT1A patients. (**F**) There were no significant correlations of plasma p62 levels with ulnar compound muscle action potentials.

We compared the levels of plasma p62 concentrations in CMT1A to CMTNSv2 to determine whether plasma p62 level could reflect the disease severity. CMT1A patients with CMTNSv2 ≥ 11 had higher median p62 concentration compared to patients with a CMTNSv2 ≤ 10 (*p* < 0.0001; Fig. 2A). The higher levels of plasma p62 concentration was maintained in CMT1A patients with CMTNSv2 ≥ 21. Pearson correlation analysis revealed that there was a significant correlation between plasma p62 level and CMTNSv2 in CMT1A patients (*r* = 0.621, *p* < 0.0001, Fig. 2B). In addition, disease duration was a positive factor correlated with plasma p62 concentration in CMT1A patients (*r* = 0.364, *p* < 0.01, Fig. 2C), whereas, onset age was negatively correlated with plasma p62 concentration (*r* =  − 0.314, *p* < 0.01, Fig. 2D).

We also compared the plasma concentration of p62 to nerve conduction velocity, an index of demyelination, in patients with CMT1A. Ulnar motor nerve conduction studies revealed a significant negative correlation between plasma p62 concentration and nerve conduction velocity in CMT1A (*r* =  − 0.490, *p* < 0.0001, Fig. 2E), but not with ulnar compound action potential (*r* =  − 0.233, *p* > 0.05, Fig. 2F**)**. In lower limb study, we could not find a meaningful correlation between the plasma p62 concentration and peroneal CMAP.

### C22 mice, a mouse model of CMT1A, exhibited the increase of p62 in Schwann cells and in plasma

**Figure 3:**
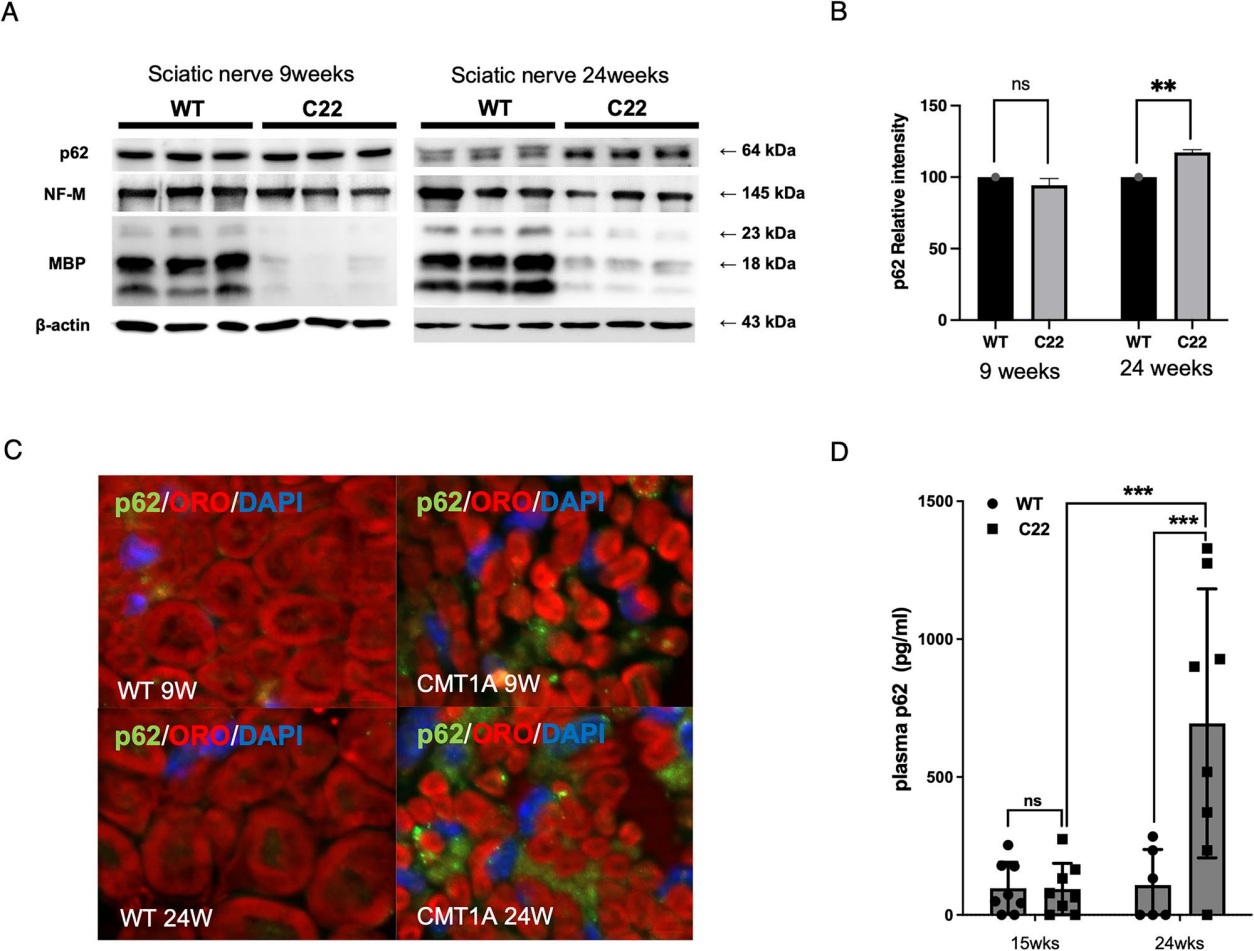
Increased p62 expression in the sciatic nerves and plasma of C22 mice. (**A**) Representative Western blots comparing the expression of p62 in the sciatic nerves of wild type (WT) and C22 mice at postnatal 9 weeks and 24 weeks. The cropped blots are used in the figure. The membranes were cut before exposure so that only a portion of the gel containing the desired bands would be visualized. NF-M; neurofilament medium-size, MBP; myelin basic protein. (**B**) Quantification of the Western blots (n = 3) (** = *p* < 0.01; ns = not significant). (**C**) Representative IF staining for p62 (green) showing several small p62 puncta in perimyelin area in C22 mice at postnatal 9 and 24 weeks. Myelin was revealed by oil-red O stain (ORO). Size bar = 10 μm. (**D**) ELISA results showing plasma concentrations of p62 in WT mice and C22 mice at postnatal 15 weeks (n = 8) and 24 weeks (n = 8). ** = *p* < 0.01, *** = *p* < 0.001).

To determine the elevation of p62 expression in SCs of a mouse model of CMT1A (the C22 mice), we examined p62 protein expression in the sciatic nerves of C22 mice using western blot analysis and immunofluorescence (IF) staining. Western blotting showed that p62 protein levels were significantly elevated in the sciatic nerves of C22 mice compared to those in wild type (WT) at 24 weeks (Fig. 3A, 3B). IF staining revealed punctate p62 stainings in the perimyelin area of myelinating SCs in the sciatic nerve of C22 mice at 9 weeks, which were further increased at 24 weeks (Fig. 3C, Supplementary Fig. 1). However, punctate p62 immunostaining was barely detected in SCs of WT mice. Next, we examined the plasma p62 levels in WT and C22 mice at two time points. ELISA revealed that the median plasma p62 level in C22 mice at 15 weeks was not significantly different from that of WT, however, it was substantially increased in C22 mice at 24 weeks after birth compared to that in WT mice at same age (*p* < 0.05, Fig. 3D).

## Discussion

We have developed blood biomarkers for peripheral neuropathy based on the concept of pathological SCs^5^. Pathological SC is a type of demyelinating SC, which is a transdifferentiated SC for active demyelination under various demyelination conditions^22^. In CMT1A, the pathological SC is an abnormally differentiated SCs that constitutes onion bulb cells and demyelinating SCs. These abnormal cells express unique markers, such as neural cell adhesion molecule 1 and growth/differentiation factor-15, and it was recently reported that such marker proteins were elevated in the blood of CMT1A patients^5,7^. It was well established that autophagy dysfunction was implicated in the pathogenesis of CMT1A^16–18^ and that autophagy receptors could be secreted when autophagic flux is dysfunctional^14,15^. Therefore, we hypothesised that the accumulation of p62, an autophagy receptor, in SCs in CMT1A patients could be reflected in their plasma. We found a comparable increase in plasma p62 levels not only in the demyelinating neuropathy of CMT1A but also in a mouse model of CMT1A, compared to that of the controls. Because ubiquitinated cargoes and p62 are accumulated in SCs of CMT1A mouse model, we suggest that dysfunction of autophagy flux leads to the accumulation of p62 in SCs and that accumulated p62 may be subsequently released into extracellular space. Thus, pursuing a pathological SC marker in patients with CMT1A is an encouraging strategy for developing blood biomarkers for demyelinating type of CMT.

It has been shown that demyelinating SCs during Wallerian demyelination after axonal injury utilize autophagy for myelin clearance^22–24^. We recently reported that demyelinating SCs in Wallerian demyelination employ autophagy machineries for excreting degenerating myelin and that p62 secretion from SCs could be an index of such demyelination activity of SCs^25,26^. Those reports further support our hypothesis of the potential SC source of plasma p62 elevation in patients with demyelinating CMT. Since demyelinating SCs have also been found in an animal model of CMTX1^27^, further studies to determine the specificity of plasma p62 elevation in demyelinating subtypes of CMT among large CMT cohorts are necessary.

In the present study, we demonstrated that plasma p62 concentration correlates with disease severity and duration in CMT1A patients, suggesting the usefulness of plasma p62 measurement for evaluating CMT1A disease activity and progression. However, accurately determining the disease duration of CMT1A patients is challenging. Plasma p62 concentration may indicate the potential for biomarker that reflect the extent of nerve damage at the specific time point. It has been shown that plasma neurofilament light chain concentration measured by Simoa test is a promising way for evaluating the disease severity for CMT^4^. However, its level is too low to accurately measure with ELISA, hindering easy and general accessibility. Thus, measuring plasma p62 concentration using ELISA may be a more useful tool for evaluating disease severity in CMT1A.

For the estimation of therapeutic trials in CMT, the development of a sensitive biomarker that reveals potential changes in neuromuscular pathology within 1 or 2 years is a major challenge^28,29^. Recent studies have employed magnetic resonance imaging (MRI) to detect chronic intramuscular fat accumulation as an index of neuropathy progression in CMT1A^30^. Interestingly, muscle MRI is sensitive enough to reflect degenerative muscle changes over a period of one year, and, thus it provides a potential outcome measurement for therapeutic trials of CMT1A^31^. It will be interesting to compare plasma p62 levels and intramuscular fat accumulation using MRI, and to determine 1 or 2 years longitudinal changes in plasma p62 concentration in CMT1A. These studies may help us develop plasma p62 measurement as an additive tool to easily assess pathological changes and treatment outcomes in CMT. In particular, autophagy activation was suggested to be a hopeful strategy to remedy demyelinating CMT, and thus the monitoring plasma p62 levels would be an appropriate tool to monitor therapeutic outcomes with pathological improvement in those trials.

In conclusion, ELISA for measuring plasma p62 might be a simple and useful tool for evaluating disease severity in the demyelinating neuropathy of CMT. Future studies are needed to examine plasma p62 levels in other forms of acquired peripheral neuropathies.

### Supplementary Information

Supplementary Figure 1.

## Data Availability

The datasets used and/or analysed during the current study available from the corresponding author on reasonable request.
