# Supplementary material for: p62/sequestosome-1 as a severity-reflecting plasma biomarker in Charcot–Marie–Tooth disease type 1A
Source: Sci Rep. 2024 May 14;14:10972. doi: 10.1038/s41598-024-61794-w (PMC11094036; doi:10.1038/s41598-024-61794-w)

p62/sequestosome-1 as a severity-reflecting plasma biomarker in Charcot-Marie-Tooth disease type 1A

**Authors**

Byeol-A Yoon, MD,^1,2#^ Young Hee Kim, PhD,^1,3#^ Soo Hyun Nam, PhD,^4^ Hye-Jin Lee, MS,^1,3^ Seong-il Oh, MD, PhD,^5^ Namhee Kim, MD,^6^ Kyeong-Hee Kim, MD,^6^ Young Rae Jo, MD, PhD,^3^ Jong Kuk Kim, MD, PhD,^1,2^ Byung-Ok Choi, MD, PhD,^4,7*^ and Hwan Tae Park, MD, PhD^1,3*^

**These authors contributed equally to this work.**

# Byeol-A Yoon, MD, Young Hee Kim, PhD:

^1^ Peripheral Neuropathy Research Center (PNRC), Department of Translational Biomedical Sciences, Graduate School of Dong-A University, Busan 49201, Republic of Korea

^2^ Department of Neurology, Dong-A University College of Medicine, Busan 49201, Republic of Korea

^3^ Department of Molecular Neuroscience and Translational Biomedical Sciences, Dong-A University College of Medicine, Busan 49201, Republic of Korea.

^4^ Department of Health Sciences and Technology, SAIHST, Sungkyunkwan University, Seoul 06351, Republic of Korea

^5^ Department of Neurology, Busan Paik Hospital, Inje University College of Medicine, Busan 47392, Republic of Korea

^6^ Department of Laboratory Medicine, Dong-A University College of Medicine, Busan 49201, Republic of Korea.

^7^ Department of Neurology, Samsung Medical Center, 81 Irwon-ro, Gangnam-gu, Seoul 06351, Korea

**Corresponding authors**

Hwan Tae Park MD, PhD,

Department of Molecular Neuroscience, Dong-A University College of Medicine,

Busan 49201, Republic of Korea

Tel +82-51-240-2636, Fax. +82-51-247-3318, E-mail: [phwantae@dau.ac.kr](mailto:phwantae@dau.ac.kr)

Byung-Ok Choi, MD, PhD

Department of Neurology, Samsung Medical Center, Seoul 06351, Republic of Korea

Tel: +82-2-3410-1296, Fax: +82-2-3410-0052, E-mail: [bochoi@skku.edu](mailto:bochoi@skku.edu)

**Supplementary Figure 1.** Representative Western blots comparing the expression of p62 in the sciatic nerves of wild type (WT) and C22 mice at postnatal 9 weeks and 24 weeks. The membranes were cut before exposure so that only a portion of the gel containing the desired bands would be visualized.


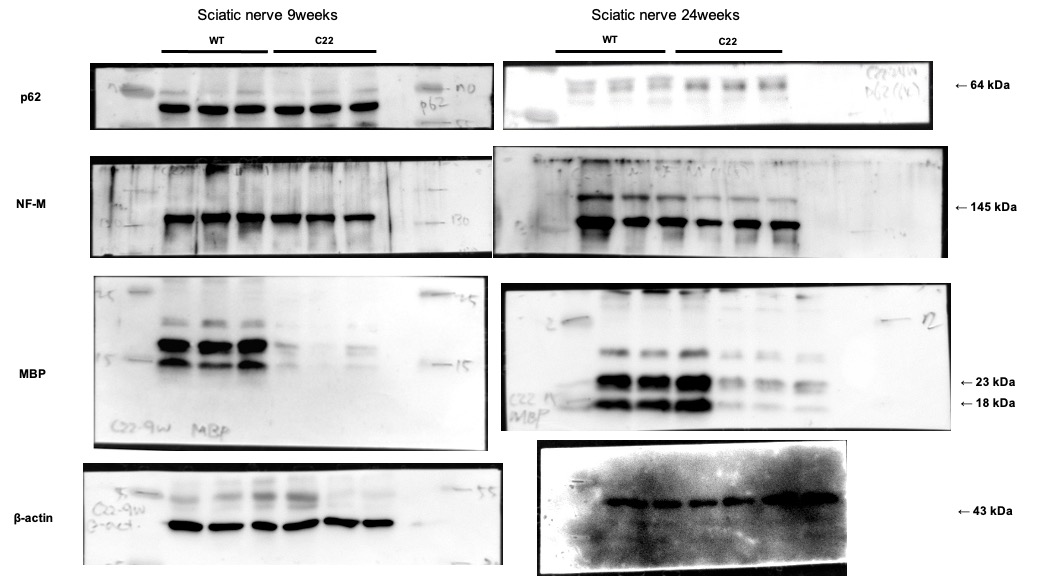

Supplement: Supplementary file 1 — Supplementary Figure 1. [file 41598_2024_61794_MOESM1_ESM.docx]
